# Patterns of Associated Knee Ligament and Chondral Injuries in First-Time Traumatic Patellar Dislocation: A Retrospective Magnetic Resonance Imaging (MRI) -Based Study

**DOI:** 10.7759/cureus.31850

**Published:** 2022-11-24

**Authors:** Fitzgerald C Anazor, Konstantinos Evangelou

**Affiliations:** 1 Trauma and Orthopaedics, Royal Berkshire Hospital, Reading, GBR

**Keywords:** magnetic resonance imaging, medial patellofemoral ligament, knee ligaments, osteochondral injury, knee trauma, patella dislocation

## Abstract

Introduction

First-time acute traumatic patellar dislocation, when managed without a knee magnetic resonance imaging (MRI) scan, may lead to missed diagnoses of important associated knee injuries. The aim of this study was to ascertain the incidence of associated ligamentous and cartilaginous injuries in first-time traumatic patella dislocation.

Methods

This was a five-year retrospective study on patients aged 16-45 who had knee MRI scans showing the characteristic bone bruise patterns seen in traumatic lateral patellar dislocation. Anonymized data from the hospital picture archiving and communication system (PACS) was obtained with each scan reviewed by a consultant radiologist, a fellowship-trained orthopaedic knee specialist, and an orthopaedic registrar or resident.

Results

A total of 200 knee MRI scans were screened. 61 eligible knee MRI scans were included in the study. The patients' ages ranged from 16 to 42 years old, with a mean of 25 years. 73.8% were male. A medial patellofemoral ligament (MPFL) tear or rupture occurred in 58 of 61 knees (95%) with MPFL attenuation in three (5%) injured knees. Meniscal injuries were identified in 5 of 61 knees (8.2%), medial collateral ligament (MCL) injuries in 11 of 61 knees (18%), osteochondral injuries and loose bodies in 17 of 61 knees (27.9%), and anterior cruciate ligament (ACL) injury in one knee (1.6%).

Conclusions

This single-centre MRI-based study has provided information on the incidence of associated chondral and ligamentous injuries in patients with first-time acute traumatic patellar dislocation. This information will be useful for clinicians when counselling patients and will add to the available literature on this injury. An MRI scan should be obtained in cases of suspected first-time traumatic patellar dislocations, especially in active young patients, due to the incidence of other associated traumatic knee lesions that might need surgical treatment and lead to persisting knee symptoms if neglected.

## Introduction

First-time traumatic patellar dislocation, also known as primary or inaugural traumatic patellar dislocation, refers to a complete displacement of the patella from the trochlear groove with an associated rupture of the previously intact medial stabilizing structures attached to the patella. Traumatic patellar dislocation accounts for 2-3% of all knee injuries [1]. These are mainly low-energy sports injuries occurring in young patients with few predisposing factors for recurrent dislocation [2,3]. These injuries result from a knee flexion and valgus injury mechanism in more than 93% of cases [4]. The lateral patellar dislocation is the most common subtype; however, medial and intra-articular subtypes are recognized but are uncommon [4]. Typical features of a first-time acute patellar dislocation on a knee magnetic resonance imaging (MRI) scan include hemarthrosis, disruption or tears of the medial patellofemoral ligament (MPFL), bone bruising in the lateral femoral condyle, and bone bruising or fracture of the medial patella facet [4,5]. First-time acute injuries of this nature, if managed without a knee MRI scan, may lead to missed diagnoses of important associated knee injuries like osteochondral and meniscal injuries that are amenable to surgical treatment.

These injuries may remain as a single event but can recur in some cases, leading to repeat dislocations. Traumatic patellar dislocation is the second most common cause of acute knee haemarthrosis after anterior cruciate ligament injuries [6]. More than 50% of patients present with persisting symptoms following a first-time traumatic patella dislocation [7]. There is a risk of future patellofemoral degeneration following a first-time traumatic patellar dislocation, along with a risk of other possible long-term sequelae like patellar instability, recurrent dislocations in 30%-40% of patients, and persistent knee pain [8-10]. Predisposing factors for progression to recurrent dislocations in these patients with a first-time injury include trochlear dysplasia, patella alta, elevated tibial tuberosity-trochlear groove distance, patella tilt, vastus medialis obliquus (VMO) hypoplasia, patella dysplasia, ligament hyperlaxity, increased femoral anteversion, and increased quadriceps angle [8,11,12].

Some hospitals treat first-time traumatic patellar dislocation injuries conservatively, with plain radiographs and closed reduction in the emergency department, and an MRI scan and referral to a knee specialist reserved for repeat dislocations or patella instability. Injuries that could potentially require surgical intervention can be missed using this approach. The aim of this retrospective study was therefore to ascertain the incidence and patterns of associated ligamentous and cartilaginous injuries on knee MRI scans performed for patients with first-time traumatic patellar dislocation presenting to our orthopaedic service. This has the potential to provide a guide for further management steps, including local decision-making on the need for MRI scans, specialist clinic follow-up, and operative treatment.

## Materials and methods

**Figure 1:**
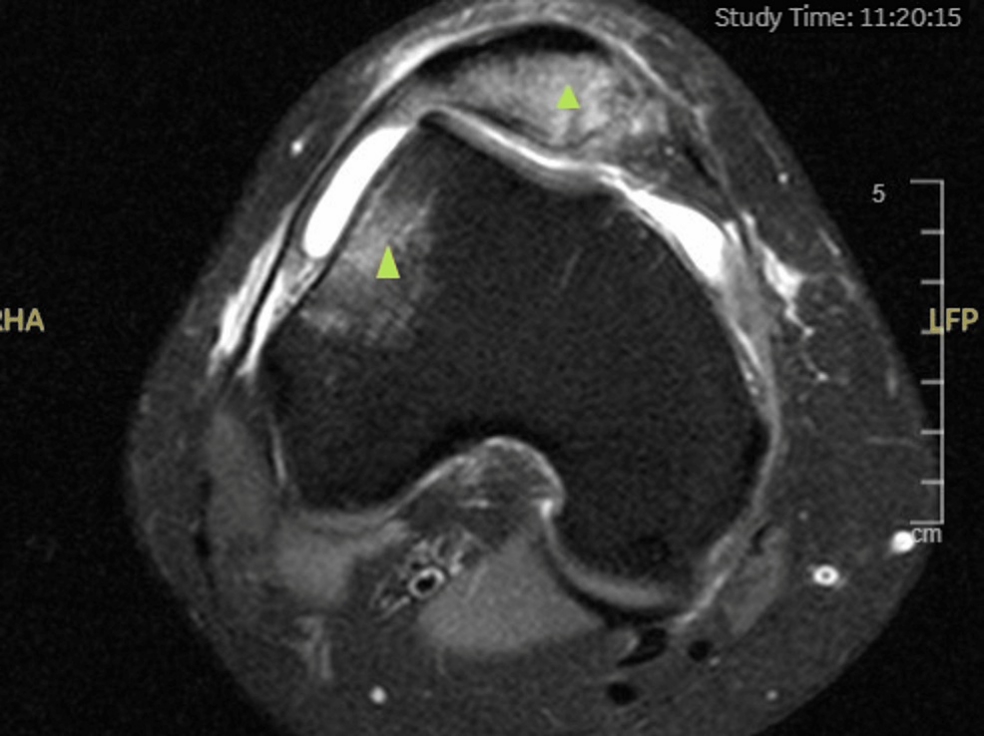
Axial T2-weighted MRI of the right knee showing the classical bone bruise pattern affecting the medial patella facet and the lateral femoral condyle (represented by the green triangles) seen in acute traumatic patellar dislocation

**Figure 2:**
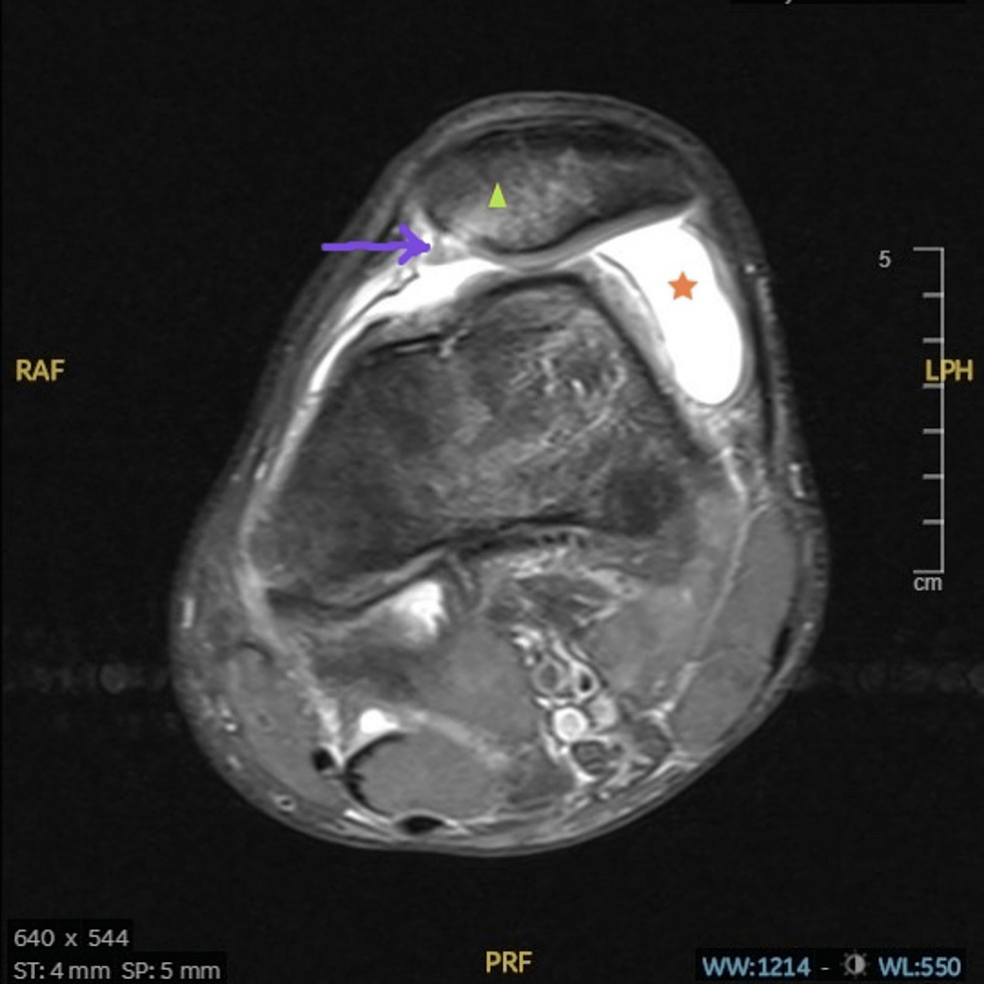
Axial T2-weighted MRI of the left knee showing medial patellofemoral ligament (MPFL) injury seen in a case of acute traumatic patella dislocation. The arrow points to the MPFL injury at the patellar insertion site; the triangle depicts the medial patellar bone bruising and the star represents the area of haemarthrosis.

This study was performed in line with the principles of the Declaration of Helsinki. The study did not involve any clinical interventions or direct involvement of human subjects. Research ethics committee approval was not required as per local guidelines. Study approval as a retrospective service evaluation project was obtained from the research and development unit in the hospital. The focus was on knee MRI scan referrals and picture archiving and communication system (PACS) reporting for first-time traumatic patellar dislocation. A five-year retrospective evaluation was performed for the period spanning from January 2016 to January 2021 for knee MRI scans (in patients aged 16-45) showing the characteristic bone bruise patterns with or without medial patella stabilising soft tissue disruption (Figures 1, 2). 

Each knee MRI scan was performed using a 1.5 Tesla high field closed Ingenia MRI system (PhillipsTM, Netherlands). Data reporting was based on anonymised images from the hospital picture archiving and communication system (PACS), with each scan reviewed by a fellowship-trained consultant radiologist as per standard reporting protocol. In addition, a fellowship-trained knee specialist (KE) and a senior orthopaedic resident (FA) also reviewed each image independently. Any missed findings from the initial radiology report individually identified by KE and FA were resolved following a joint imaging review and discussion with a musculoskeletal consultant radiologist if required. The clinical indication for each scan and any previous ipsilateral knee MRI scans available on the PACS were screened to exclude patients who had suffered a previous patella dislocation, had a history of patellar instability or met any of the other exclusion criteria (Table 1).

**Table 1:** Inclusion and exclusion criteria for this study

INCLUSION CRITERIA	EXCLUSION CRITERIA
Young patients (16-45 years of age)	Age <16 years or greater than 45 years
First-time patella dislocation from the provided clinical history	Clinical history suggestive of recurrent dislocation
MRI scan showing the typical bone bruise pattern of an acute traumatic lateral patella dislocation	MRI scan lacking the typical bone bruise pattern of an acute traumatic patella dislocation
Closed, isolated injuries	Open injuries or polytrauma

## Results

A total of 200 knee MRI scans performed for suspected traumatic patellar dislocation during the study period were screened. Of these, 61 eligible knee MRI scans were included in the study and analyzed. Only the lateral subtype of patellar dislocation was present in all of the knees studied. The mean age of the patients was 25 years (SD 6.72), ranging from 16-42 years. 45/61 (73.8%) of the patients were male. The right knee was involved in 32 (52.4%) MRI scans. MPFL tears or ruptures were seen in 58 of 61 knees (95%) with MPFL attenuation in the remaining three (5% of the injured knees). Meniscal injuries were identified in 5 of 61 knees (8.2%), with two involving the medial meniscus; medial collateral ligament (MCL) injuries in 11 of 61 knees (18%); and osteochondral injuries or loose bodies in 17 of 61 knees (27.9%). There was one anterior cruciate ligament tear in a 19-year-old patient who also had an MPFL and a grade 2 MCL tear. A comparison of each associated injury pattern with limb laterality, the mean age of the patients, and gender was also performed (Table 2).

**Table 2:** Incidence of each associated knee injury with the mean age of the patients, limb laterality and gender. MPFL= medial patellofemoral ligament; MCL= medial collateral ligament; LCL= lateral collateral ligament; ACL= anterior cruciate ligament; SD= standard deviation; N/A= not applicable

	MPFL tear/disruption	MPFL attenuation	Meniscal tear	Osteochondral injury	MCL injury	LCL injury	ACL injury
Number of knees affected	58	3	5	17	11	0	1
Laterality (right knee)	31	2	3	8	3	N/A	0
Mean age in years (SD)	24.88 (6.91)	25.67 (3.21)	29.40 (6.95)	23.56 (5.96)	26.09 (8.94)	N/A	19
Males (%)	43 (74%)	2 (67%)	5 (100%)	10 (59%)	10 (91%)	N/A	1 (100%)

Further analysis of the results showed that eight (13.1%) knees had a tibial tuberosity-trochlear groove (TT-TG) distance greater than 20 mm; 30 (49.1%) knees had a TT-TG distance between 15-20 mm; 15 (24.6%) knees had a tibial tuberosity to trochlear groove (TT-TG) distance between 10-14 mm; and eight (13.1%) knees had a TT-TG < 10 mm. 45 (73.7%) knees had a trochlear groove or sulcus angle >145 degrees, while seven (11%) knees had a patella-trochlear index (PTI) of <0.28.

## Discussion

The sensitivity of an MRI for diagnosing acute patellar dislocation is as high as 95% [13]. Another study by Zhang et al. found a sensitivity of 95% and specificity of 80%-81% for MRI in diagnosing partial or complete MPFL tears [14]. Osteochondral fractures can be missed in 30%-40% of plain radiographs done in patients with first-time patellar dislocation, and these can be readily diagnosed on an MRI scan or during diagnostic arthroscopy [15]. This emphasises the importance of obtaining an MRI scan as soon as possible in patients suffering from a first-time traumatic patellar dislocation. 

In a study by Guerrero et al. involving 195 patients with a first-time traumatic patellar dislocation, the mean age of the patients was 23 years [16]. In another study by Sanders et al. involving 609 patients, the mean age of the patients was 21.4 +/-9.9 years [17]. These figures are comparable to the mean age of 25 years in this study population, which is in keeping with the fact that most of these injuries occur in the young, active population in their third decade of life. There was no significant variation in mean age for the different associated injuries in this study. In a case series performed by Petri et al. involving 40 patients, the right knee was involved in 44% of cases [18]. On the other hand, the right knee was involved in 52% of our knee MRI scans. There is mixed evidence in the literature on the male-to-female ratio for first-time traumatic patellar dislocation. In a systematic review by Stefancin et al., 54% of the patients were female [1]. On the other hand, 35% of the patients were female in the study by Guerrero et al., which is similar to the findings from our study [16]. 

Previous knee MRI-based studies have shown that MPFL disruption occurs in 90%-100% of acute traumatic patellar dislocations [19-21]. These results are similar to the findings from this study, where 95% of the injured knees had an MPFL tear or disruption. Guerrero et al. reported MPFL injuries or attenuation in 99% of the studied knees; osteochondral injuries with or without loose body injuries in 62%; meniscal injuries in 21%; and MCL injuries in 19% of the studied knees [16]. Sillanpää et al. found associated MPFL injuries in 100%, osteochondral injuries in 44%, meniscal injuries in 4%, and ACL injuries in 1% of the injured knees [4]. Our study showed an incidence of osteochondral injuries in 27% of knees, meniscal injuries in 8%, MCL injuries in 18%, and ACL injuries in 1.6% of knees. These figures show a wide variation of associated osteochondral, chondral, and ligament injuries in various studies. However, the results show that osteochondral injuries are the second most common associated injury (after MPFL tears) in first-time traumatic patellar dislocation, while meniscal injuries are the third most common associated injuries. The MCL is injured in some cases, with lateral collateral ligament (LCL) injuries being rare in keeping with the injury mechanism.

In this study, 28% of the knees had a borderline or elevated TT-TG (> 15 mm) on MRI imaging. The normal TT-TG distance on MRI is 10 +/- 1 mm, with a wide variation reported in the literature. Any value above 20 mm is considered pathological, while 15-20 mm is borderline [22-24]. The normal trochlear groove (sulcus) angle is 130-145 degrees, with angles > 145 degrees predisposing to recurrent patella dislocation. This study showed that 74% of the knees had an abnormal sulcus angle, and these patients have an increased risk of recurrent dislocation in the future. Furthermore, Fones et al. found a statistically significant correlation between an abnormal sulcus angle and the incidence of associated osteochondral pathology [25]. This is similar to the findings in our study, where 14 (82%) knees with associated osteochondral pathology had an increased sulcus angle > 145 degrees. In addition, PTI < 0.28 is considered pathologic [26]. In our study, an abnormal PTI was found in 11% of the knees, which can predispose to recurrent dislocations. 

A study by Martinez-Cano et al. showed an additive effect of patella alta, trochlear dysplasia, and age less than 21 years for predicting the risk of recurrent dislocation following a first-time traumatic patellar dislocation [27]. They calculated the predicted probability of dislocation in the presence of no factor as 31%, one factor as 36%, two factors as 71%, and a risk of 86% in the presence of three factors. In our study, 34% of the patients had at least two of these factors, while 5% had three factors. Thus, there is a need to get an MRI to identify these risk factors and also follow up on these patients due to the risks of recurrent dislocation, even in first-time traumatic patellar dislocation.

The systematic review by Stefancin et al. comparing non-operative treatment and operative treatment recommended non-operative treatment for first-time traumatic patellar dislocation unless there is clinical and/or radiographic evidence of osteochondral fractures, chondral injuries, or large medial patella stabilising defects (medial retinaculum, MPFL, VMO) [1]. Another systematic review by Longo et al. comparing the same treatment modalities for this injury showed a higher re-dislocation rate (36.4%) in the non-operatively treated knees compared to the knees that had surgical intervention (25%) [28]. However, they could not provide a definitive treatment recommendation due to the fact that the included studies (including the identified randomized control trials) were of low quality with a high risk of bias from small sample sizes, while other studies lacked randomisation and/or blinding.

This study had certain strengths. There were clear inclusion and exclusion criteria. All consecutively identified knee scans that met the inclusion criteria were included. The inter-observer error was minimised by having three clinicians (including a fellowship-trained consultant radiologist) review each scan. The intra-observer error was minimised by KE and FA reviewing each individual scan twice on two different occasions following initial reporting by a consultant radiologist. The study has analysed the association between the various associated chondral and ligament injuries and the mean age of patients and injury laterality. It has also involved an analysis of the risk factors for future re-dislocation as seen on knee MRI scans in these patients.

Limitations

This study had some limitations. It did not involve direct clinical history-taking or the performance of any clinical examination on patients, as this was mainly an anonymized PACS data and image-based retrospective evaluation. Correlating the clinical findings with the imaging findings could have provided more information for each patient. In addition, no follow-up for the patients with the identified risk factors for recurrent dislocation was completed as part of this study. Finally, a larger sample size could have increased the statistical power of the findings from this study.

## Conclusions

This single-centre MRI-based study has provided information on the incidence of associated chondral and ligamentous injuries in patients with first-time acute traumatic patellar dislocation. This information will be useful for clinicians when counselling patients and will add to the available literature on this injury. This study has shown that patients in our local practice with a clinical diagnosis of first-time traumatic patellar dislocation will benefit from an MRI scan as soon as is feasible following the initial closed reduction in order to identify these associated chondral, osteochondral, and ligamentous injuries that can increase the risk of future dislocations, instability, and persistent symptoms. This is especially important as the patients who are affected by these injuries are mainly young and very active. These injuries may require surgical intervention and should prompt a referral to a knee specialist once identified.

Local trauma and orthopaedic guidelines will be modified in the hospital in response to the findings from this study. In all patients with a clinical diagnosis of first-time traumatic patellar dislocation, these include an orthopaedic review in the emergency department prior to discharge, obtaining a knee MRI scan, and referring to a specialist knee clinic as soon as possible. 
